# Supplementary material for: Determinants of prelabor rupture of membrane among pregnant women attending governmental hospitals in Jimma zone, Oromia region, Ethiopia: A multi-center case-control study
Source: PLoS One. 2023 Nov 30;18(11):e0294482. doi: 10.1371/journal.pone.0294482 (PMC10688638; doi:10.1371/journal.pone.0294482)
Supplement: S1 Checklist — (DOCX) [file pone.0294482.s001.docx]

STROBE checklist for the study “Determinants of prelabor rupture of membrane among pregnant women attending governmental hospitals in Jimma zone, Oromia region, Ethiopia: A multi-center case control study”

|  | Item No. | Recommendation | Page  No. |
| --- | --- | --- | --- |
| **Title and abstract** | 1 | (*a*) Indicate the study’s design with a commonly used term in the title or the abstract | 1 |
|  |  | (*b*) Provide in the abstract an informative and balanced summary of what was done and what was found | 2, 3 |
| Introduction | | | |
| Background/rationale | 2 | Explain the scientific background and rationale for the investigation being reported | 4-6 |
| Objectives | 3 | State specific objectives, including any prespecified hypotheses | 6 |
| Methods | | | |
| Study design | 4 | Present key elements of study design early in the paper | 6 |
| Setting | 5 | Describe the setting, locations, and relevant dates, including periods of recruitment and data collection | 6-8 |
| Participants | 6 | *Case-control study*—Give the eligibility criteria, and the sources and methods of case ascertainment and control selection. Give the rationale for the choice of cases and controls | 8 |
|  |  |  |  |
| Variables | 7 | Clearly define all outcomes, exposures, predictors, potential confounders, and effect modifiers. Give diagnostic criteria, if applicable | 9,10 |
| Data sources/ measurement | 8* | For each variable of interest, give sources of data and details of methods of assessment (measurement). Describe comparability of assessment methods if there is more than one group | *9,10* |
| Bias | 9 | Describe any efforts to address potential sources of bias | 10 |
| Study size | 10 | Explain how the study size was arrived at | 8,9 |

Continued on next page

| Quantitative variables | 11 | Explain how quantitative variables were handled in the analyses. If applicable, describe which groupings were chosen and why | 11 |
| --- | --- | --- | --- |
| Statistical methods | 12 | Describe all statistical methods | 11 |
| Participants | 13* | (a) Report numbers of individuals at each stage of study—eg numbers potentially eligible, examined for eligibility, confirmed eligible, included in the study, and analysed | 13 |
| Descriptive data | 14* | (a) Give characteristics of study participants (eg demographic, clinical, social) and information on exposures and potential confounders | 11 |
|  |  | *Case-control study—*Report numbers in each exposure category, or summary measures of exposure | *11-18* |
| Main results | 16 | (*a*) Give unadjusted estimates and, if applicable, confounder-adjusted estimates and their precision (eg, 95% confidence interval). Make clear which confounders were adjusted for and why they were included | 18-20 |
|  |  | (*b*) Report category boundaries when continuous variables were categorized | 18-20 |

Continued on next page

| Key results | 17 | Summarise key results with reference to study objectives 21 |  |
| --- | --- | --- | --- |
| Limitations | 18 | Discuss limitations of the study, taking into account sources of potential bias or imprecision. Discuss 24 both direction and magnitude of any potential bias |  |
| Interpretation | 19 | Give a cautious overall interpretation of results considering objectives, limitations, multiplicity of analyses, results from similar studies, and other relevant evidence | 21-24 |
