# Supplementary material for: Determinants of prelabor rupture of membrane among pregnant women attending governmental hospitals in Jimma zone, Oromia region, Ethiopia: A multi-center case-control study
Source: PLoS One. 2023 Nov 30;18(11):e0294482. doi: 10.1371/journal.pone.0294482 (PMC10688638; doi:10.1371/journal.pone.0294482)
Supplement: Attachment — Submitted filename: Response to Reviewers.docx [file pone.0294482.s004.docx]

**Rebuttal letter**

Dear editor Dr. Kahsu Gebrekidan, and dear reviewers,

We would like to thank you for giving us the opportunity to submit a revised version of our manuscript titled “*Determinants of prelabor rupture of membrane among pregnant women attending governmental hospitals in Jimma zone, Oromia region, Ethiopia: A multi-center case control study”*. We are too grateful to you and the reviewers for taking the time and effort to read our manuscript and offer insightful comments. Based on these comments and suggestions, we have made careful modifications to the original manuscript. Your informed and helpful feedback helped us think of potential changes for the current version. We believe the manuscript has improved and hope that it meets your high standards. The authors welcome further constructive comments, if any.

Please see below for a point-by-point response to comments and concerns. Comments are shown in black, followed by our answer/responses in dark blue. The changes are shown in the marked copy of the manuscript (highlighted in blue).

Best regards,

**Academic Editor’s Comment**

Dear Dr. Assefa,

Thank you for submitting your manuscript to PLOS ONE. After careful consideration, we feel that it has merit but does not fully meet PLOS ONE’s publication criteria as it currently stands. Therefore, we invite you to submit a revised version of the manuscript that addresses the points raised during the review process.

Please revise the manuscript based on the feedback from reviewers.

- The manuscript needs English revision

**Response**: We have revised the manuscript thoroughly accordingly.

- Please follow the PLOS ONE submission guideline, for example on page 6, the title should be Methods and materials

**Response**: We have revised the manuscript based on the PLOS ONE submission guideline.

Please include the following items when submitting your revised manuscript:

- A rebuttal letter that responds to each point raised by the academic editor and reviewer(s). You should upload this letter as a separate file labeled 'Response to Reviewers'.
- A marked-up copy of your manuscript that highlights changes made to the original version. You should upload this as a separate file labeled 'Revised Manuscript with Track Changes'.
- An unmarked version of your revised paper without tracked changes. You should upload this as a separate file labeled 'Manuscript'.

If you would like to make changes to your financial disclosure, please include your updated statement in your cover letter. Guidelines for resubmitting your figure files are available below the reviewer comments at the end of this letter.

If applicable, we recommend that you deposit your laboratory protocols in [protocols.io](http://protocols.io/) to enhance the reproducibility of your results

Journal requirements:

When submitting your revision, we need you to address these additional requirements.

1. Please ensure that your manuscript meets PLOS ONE's style requirements, including those for file naming.

**Response:** We agree with this and have incorporated your suggestion throughout the revised manuscript.

2. We suggest you thoroughly copyedit your manuscript for language usage, spelling, and grammar.

**Response:** Thank you for this suggestion. We did our best to copyedit our manuscript for language usage, spelling, and grammar.

3. We note that Figure 1 in your submission contain [map/satellite] images which may be copyrighted. All PLOS content is published under the Creative Commons Attribution License (CC BY 4.0), which means that the manuscript, images, and Supporting Information files will be freely available online, and any third party is permitted to access, download, copy, distribute, and use these materials in any way, even commercially, with proper attribution. For these reasons, we cannot publish previously copyrighted maps or satellite images created using proprietary data, such as Google software (Google Maps, Street View, and Earth).

We require you to either (1) present written permission from the copyright holder to publish these figures specifically under the CC BY 4.0 license, or (2) remove figures from your submission.

**Response:** Thank you for pointing this out. Since it is difficult to get written permission from the copyright holder, the authors agree to remove Figure 1 from our submission. Therefore, Figure 1 has now been removed from the revised manuscript.

**Reviewer reports**

**Reviewer #1: Dear author**

This is a very interesting paper, nice and easy to read.

I wish you used another word to replace the word predictors! or determinants! It would be perfect since you only measured associations and not risk ratio. Measures of associations only give associations and not causal relations; the predictors and determinants seem more like a causal relation.

**Response:** The authors thank the reviewer for his kind words. We very much appreciate the reviewer’s detailed evaluations and suggestions. We agree with most of them, and the manuscript has been revised thoroughly according to the reviewer’s advice. The authors have chosen to use the word determinants in the title of the study because we have used a case control study design in our study. Analytic studies focus on elucidating the determinants of disease. During the case control study design, we determined what proportion of cases were exposed and what proportion were not. We have also determined what proportion of controls were exposed and what proportion were not. Hence, the aim of case-control studies is to identify the determinants/predictors/risk factors of the disease. Furthermore, many case control studies that are published in peer-reviewed journals use those words. Due to those reasons, the authors preferred the word determinants in this study.

I would think that adding line numbers to the manuscript would make it easy to tract the comments.

**Response:** We thank the reviewer for this good suggestion. We have added line numbers to the revised manuscript.

Results: Table 5. is a combination of bivariable and multivariable findings, why don’t you separate the two tables and report them independently?

**Response:** We appreciate these comments. Indeed, bi-variable logistic regression was done and variables with a p-value of less than 0.25 were transported to the multivariable logistic regression model using the backward stepwise (likelihood ratio) method. The authors feel that it is enough to present and report variables that had an association with PROM in the bi-variable analysis and transferred into a multi-variable logistic regression model only (in one table). We also feel that it is not important to report all variables in bi-variable analysis using a separate table.

Study limitations: what did you do to mitigate the limitation of recall bias? Please state it clearly as a delimitation.

**Response:** Thanks for your kind question. During the data collection to reduce recall bias, we have tried to make sure that participants understand what is being asked before they respond so they don’t misinterpret it as something else and answer incorrectly based on that misunderstanding instead. We have also tried to gain some information from the hospital records, along with the patient’s history of past events.

Conclusion: does your study have something to recommend for training? It would be nice to say something on practice and training, next.

**Response:** Thank you for your comment. We agree and have now included it as suggested in the revised manuscript. It is also recommended to provide training for pregnant women to improve awareness and to take preventive measures for the determinants of PROM.

Be specific about what you would like the readers to study

**Response:** Thank you for noticing. Based on the findings of our study, we have added recommendation for the readers which is presented as follow in the revised manuscript**:** In a future study, it would be interesting to study factors that may have association with PROM, such as periodontal disease and micronutrient deficiency**.**

**Reviewer #2: Mesfin Abebe**

Title: Determinants of prelabor rupture of membrane among pregnant women attending governmental hospitals in Jimma zone, Oromia region, Ethiopia: A multi-center case control study

Thank you for the opportunity to review this manuscript I read the manuscript in detail and the manuscript is well-structured and written in a good manner. However, I raised some major comments and suggestions below. I'm hopeful the comments below can help fill these gaps and improve the quality of the paper.

Major revision

The paper's overall language quality is a little low; there are numerous grammatical errors throughout the entire manuscript. Before the next submission, I recommend that the entire manuscript be revised by a native English speaker.

**Response:** Thank you for your thorough review and salient observations. We have tried to correct grammatical errors throughout the entire manuscript.

Abstract:

1. The abstract accurately reflects the details presented in the manuscript's body. However, the use of abbreviations/acronyms in the abstract section is not recommended, and I recommend revising it.

**Response:** Thank you for pointing this out. We agree with this comment, which has now been amended in the revised abstract.

2. You stated as in abstract section “Therefore, hospitals should give focus to the early screening, diagnosis, and treatment of modifiable risk factors to reduce the burden of prelabor rupture of membrane” What are those modifiable risk factors? Better to list those modifiable risk factors.

**Response:** The authors thank you for your interesting comment. We have changed the text following your suggestion by listing those modifiable risk factors. The text was changed as follows: “Therefore, hospitals should give focus to the early screening, diagnosis, and treatment of pregnancy induced hypertension, urinary tract infection, and abnormal vaginal discharge to reduce the burden of prelabor rupture of membrane.”

Background:

1. Your background information is somewhat good, but didn’t show a gap in your study. The author only identified as fetal sex and no prior study conducted in Jimma zone as gap. You need to write more than this.

**Response:** The authors would like to thank the reviewer for raising this point. Indeed, we tried our best to show a gap in the introduction section. This point has been presented in the manuscript as follows: Even though some research has been done in Ethiopia, the prevalence of PROM varied significantly between settings, ranging from 1.4% to 23.5%, possibly due to variations in determinants from place to place. Most of these studies used cross-sectional study designs and retrospective methods, covering small-scale areas (single-centered) as well as focusing on maternal and perinatal outcomes, and respondents with term pregnancy (above 37 weeks) were excluded.

The accurate cause of PROM remains uncertain. Despite the presence of a few studies using case-control study design, there are some conflicting or discrepant results on some determinants for PROM such as smoking, wealth index, and PIH which demands further exploration. In addition, some fetal factors like fetal sex were not assessed. Lastly, there was no study conducted in our study area regarding determinants of PROM among pregnant women.

2. You stated as “Therefore, this study aimed to address the identified gaps and examine the determinants of PROM using a case-control study among pregnant women attending governmental hospitals in the Jimma zone, Oromia region, Ethiopia.” Rewrite this sentence as “Therefore, this study aimed to identify the determinants of PROM using a case-control study among pregnant women attending governmental hospitals in the Jimma zone, Oromia region, Ethiopia.”

**Response:** Thank you very much for your suggestion. We found your comment is helpful and has now been written clearly as suggested.

Methods:

1. Better to remove the word prospective. “An institutional-based unmatched case-control study design was employed” is enough.

**Response:** Thanks for your kind reminders. We found your comment helpful and the word prospective has been removed accordingly. Therefore, this has now been stated as “An institutional-based unmatched case-control study design was employed” in the revised manuscript.

2. Why were consecutive sampling technique used to select study participants? Why not used systematic sampling technique?

**Response:** Thanks for your kind question. We appreciate the reviewer’s comments regarding sampling technique. Since we did not have a list of study participants (sampling frame), it is difficult to use systematic sampling technique. Moreover, in pregnant women attending hospitals, it is not possible to predict in advance who will be attending. Due to this reason, the eligible case was selected consecutively and the three consecutive controls were selected until the determined sample size was met during the data collection period.

3. During selection of candidate variable for final model or multivariable logistic regression you only used p value less than 0.25. Why not consider another mechanism? Because you only consider p value you may be loss some important variable. So, consider other mechanisms in addition to p value.

**Response:** Thank you for pointing this out. Existing theory and literature, as well as experience and clinical knowledge, show that the actual variables used in the final model should be determined by analyzing the data. Determining the set of variables for the final model is called variable selection. Variable selection serves two purposes. First, it helps to determine all of the variables that are related to the outcome, which makes the model complete and accurate. Second, it helps to select a model with few variables by eliminating irrelevant variables that decrease the precision and increase the complexity of the model. Ultimately, variable selection provides a balance between simplicity and fit.

It has also been suggested that variables that show significance (p<0.25) in the bivariate analysis should be included for multivariate analysis. In our study we used only the p value because it is an objective value that helps to decrease irrelevant variables, and we also included many variables for the final model. We also feel it is not appropriate to include those variables based on clinical importance or other criteria other than the p value. Furthermore, we also appreciate your further suggestions, if any.

Results

1. In table 1; what is your reference for category of average monthly income?

**Response:** Thank you for raising this point. Since we have collected data regarding average monthly income only in the form of cash (Ethiopian Birr), it is difficult to create the wealth index. Because creating the wealth index requires selecting variables (such as productive, non-productive assets, household assets and others) to be incorporated in the questionnaire. Due to this reason, we have only chosen to categorize the average monthly income based on previous researches that have been published in the peer reviewed journal.

*“Assefa NE, Berhe H, Girma F, Berhe K, Berhe YZ, Gebreheat G et al. Risk factors of prelabor rupture of membranes in public hospitals at Mekele city, Tigray, a case control study. BMC pregnancy childbirth. 2018;18(1):1–7”*

*“Gerema et al. Determinants of ectopic pregnancy among pregnant women attending referral hospitals in southwestern part of Oromia regional state, Southwest Ethiopia: a multi-center case control study. BMC Pregnancy and Childbirth. 2021; 21:130”*

2. What is the difference between Nulliparity and Primipara?

**Response:** Thanks for your kind question. A female who has not given birth who reached viability is called a nullipara or para 0, whereas a female who has given birth once is primipara or para 1.

Discussion:

1. The discussion is generally well-organized and incorporates existing literature. There might be more consideration given to the next steps in the research or implementation.

**Response:** We are grateful to the reviewer for these kind suggestions. The discussion has been revised thoroughly according to the reviewer’s advice.

2. You stated “recall and social desirability bias as limitation of your study” so what mechanism used to minimize those bias? Because of those bias common in case control study design. Please write down how to minimize those bias beside of limitation of study.

**Response:** Thanks for your kind question. This question was also addressed by the other reviewer and, similar to the previous response, is addressed in the text: During the data collection to reduce recall bias, we have tried to make sure that participants understand what is being asked before they respond so they don’t misinterpret it as something else and answer incorrectly based on that misunderstanding instead. We have also tried to gain information from the hospital records along with the patient’s history of past events. We have also tried to allow anonymity and guarantee full confidentiality for the study participants to reduce social desirability bias. In addition, training was also provided for data collectors regarding how to approach study participants especially related to sensitive behaviors.

3. In abbreviation section I was see some acronyms. So, rewrite as abbreviations and acronyms

**Response:** Thank you for noticing. We have revised as abbreviations and acronyms.
