# Supplementary material for: Determinants of prelabor rupture of membrane among pregnant women attending governmental hospitals in Jimma zone, Oromia region, Ethiopia: A multi-center case-control study
Source: PLoS One. 2023 Nov 30;18(11):e0294482. doi: 10.1371/journal.pone.0294482 (PMC10688638; doi:10.1371/journal.pone.0294482)
Supplement: Attachment — Submitted filename: Reviewer comments for determinants of PROM.docx [file pone.0294482.s003.docx]

Reviewer reports

Title: Determinants of prelabor rupture of membrane among pregnant women attending governmental hospitals in Jimma zone, Oromia region, Ethiopia: A multi-center case control study

Reviewer: Mesfin Abebe

Thank you for the opportunity to review this manuscript I read the manuscript in detail and the manuscript is well-structured and written in a good manner. However, I raised some major comments and suggestions below. I'm hopeful the comments below can help fill these gaps and improve the quality of the paper.

Major revision

The paper's overall language quality is a little low; there are numerous grammatical errors throughout the entire manuscript. Before the next submission, I recommend that the entire manuscript be revised by a native English speaker.

Abstract:

1. The abstract accurately reflects the details presented in the manuscript's body. However, the use of abbreviations/acronyms in the abstract section is not recommended, and I recommend revising it.
2. You stated as in abstract section “Therefore, hospitals should give focus to the early screening, diagnosis, and treatment of modifiable risk factors to reduce the burden of prelabor rupture of membrane” What are those modifiable risk factors? Better to list those modifiable risk factors.

Background:

1. Your background information is somewhat good, but didn’t show a gap in your study. The author only identified as fetal sex and no prior study conducted in jimma zone as gap. You need to write more than this.
2. You stated as “Therefore, this study aimed to address the identified gaps and examine the determinants of PROM using a case-control study among pregnant women attending governmental hospitals in the Jimma zone, Oromia region, Ethiopia.” Rewrite this sentence as “Therefore, this study aimed to identify the determinants of PROM using a case-control study among pregnant women attending governmental hospitals in the Jimma zone, Oromia region, Ethiopia.”

Methods:

1. Better to remove the word prospective. “An institutional-based unmatched case-control study design was employed” is enough.
2. Why were consecutive sampling technique used to select study participants? Why not used systematic sampling technique?
3. During selection of candidate variable for final model or multivariable logistic regression you only used p value less than 0.25. Why not consider another mechanism? Because you only consider p value you may be loss some important variable. So consider other mechanisms in addition to p value.

Results

1. In table 1; what is your reference for category of average monthly income?
2. What is the difference between Nulliparity and Primipara?

Discussion:

1. The discussion is generally well-organized and incorporates existing literature. There might be more consideration given to the next steps in the research or implementation
2. You stated “recall and social desirability bias as limitation of your study” so what mechanism used to minimize those bias? Because of those bias common in case control study design. Please write down how to minimize those bias beside of limitation of study.
3. In abbreviation section I was see some acronyms. So rewrite as abbreviations and acronyms
